# Supplementary figures and images for: Sponge symbiosis is facilitated by adaptive evolution of larval sensory and attachment structures in barnacles
Source: Proc Biol Sci. 2020 May 13;287(1927):20200300. doi: 10.1098/rspb.2020.0300 (PMC7287368; doi:10.1098/rspb.2020.0300)

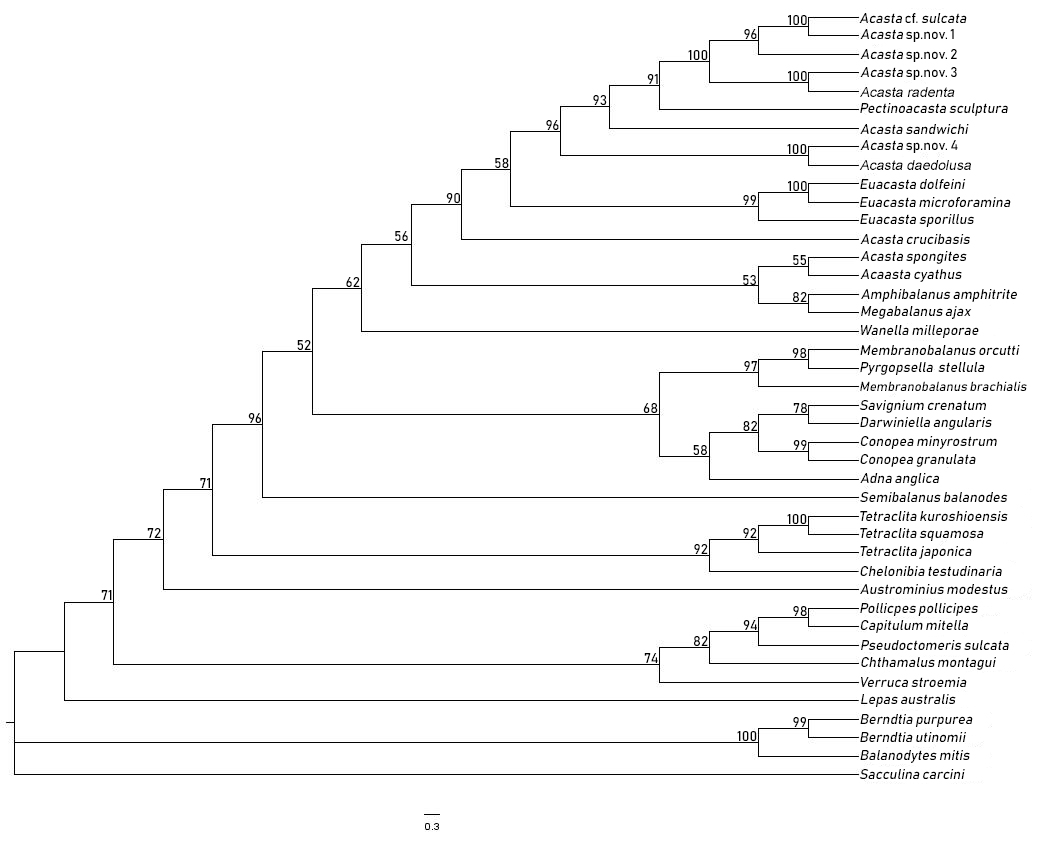

Supplement: Figure S1. ML tree [file rspb20200300supp1.jpg]

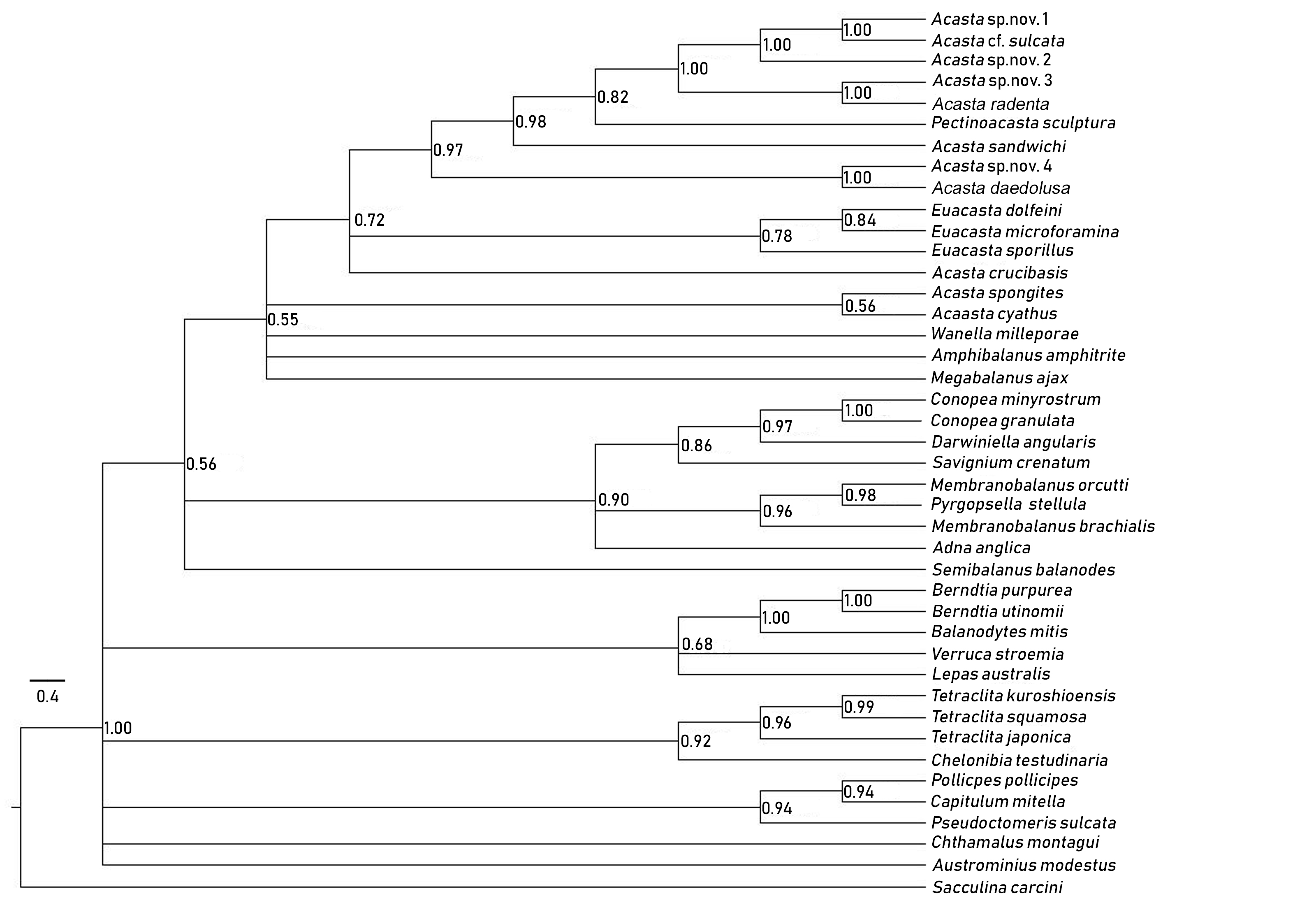

Supplement: Figure S2. BI tree [file rspb20200300supp2.jpg]

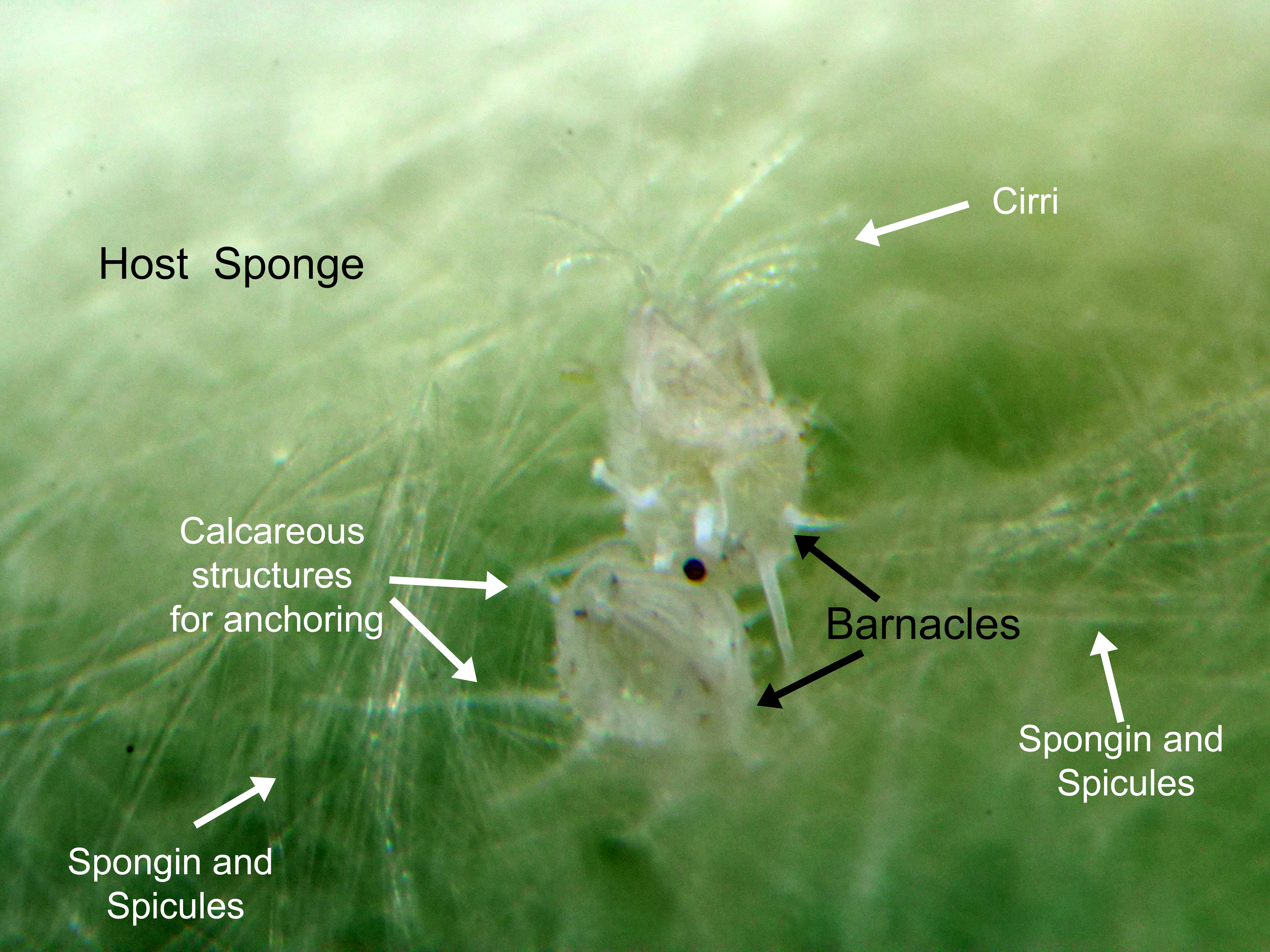

Supplement: Figure S3. Ed anchoring [file rspb20200300supp3.jpg]

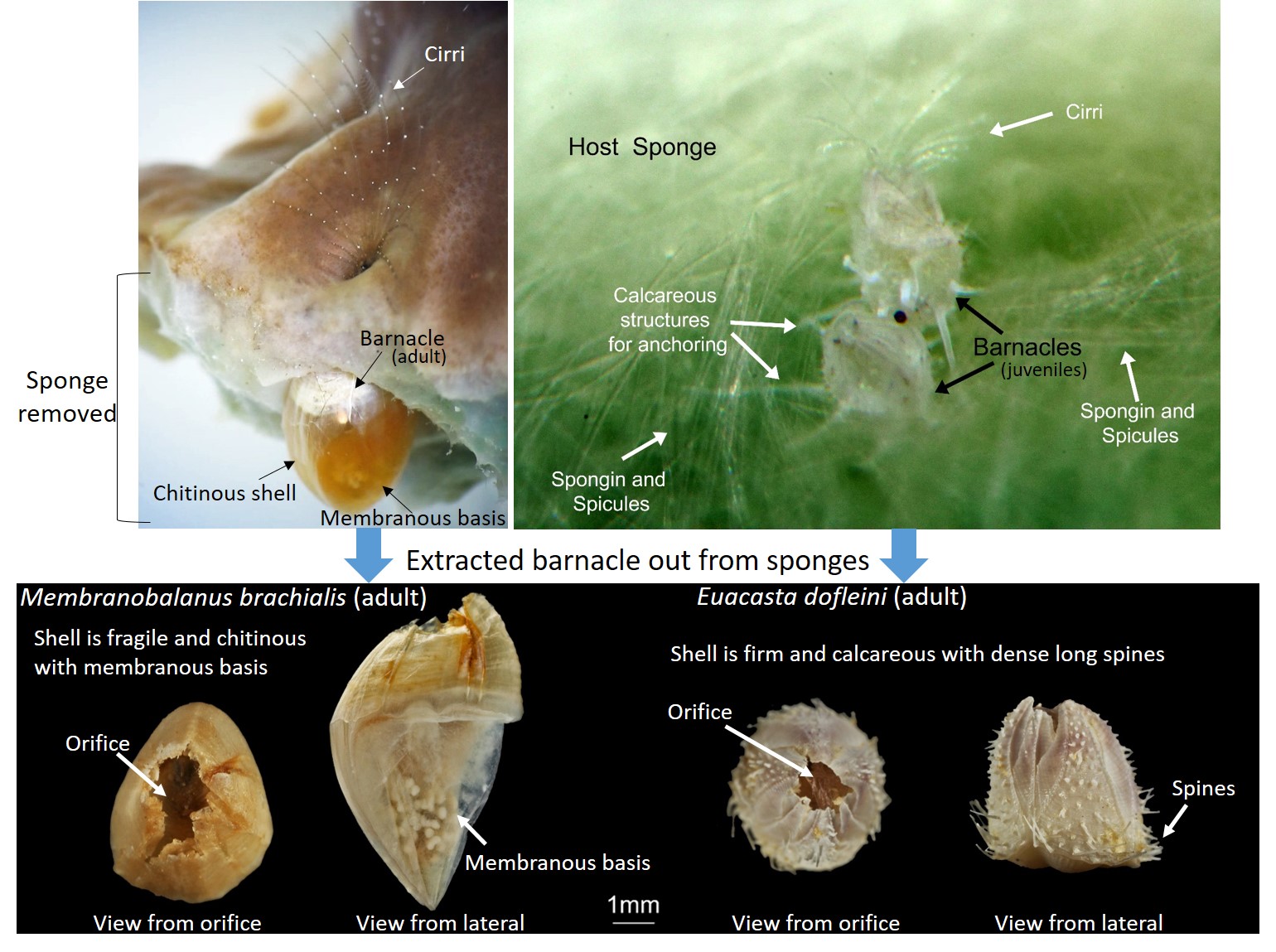

Supplement: Figure S4. Shell comparison [file rspb20200300supp4.jpg]
