# Supplementary material for: Sponge symbiosis is facilitated by adaptive evolution of larval sensory and attachment structures in barnacles
Source: Proc Biol Sci. 2020 May 13;287(1927):20200300. doi: 10.1098/rspb.2020.0300 (PMC7287368; doi:10.1098/rspb.2020.0300)
Supplement: Paragraph S1a-d. Materials and Methods [file rspb20200300supp6.docx]

**Paragraph 1_Materials and methods**

**a. Collection and cultivation.**

Sponge barnacles and host sponges were collected in Taiwan, South Korea and Japan by SCUBA diving at depths of 3-20 m. Sponge barnacles and host sponges were maintained in lab setting aquarium. Adult sponge barnacles were separated from the host sponge and cultured in 500 ml beakers. Nauplii were collected after being released from the adults and reared into cyprids. All species have six naupliar instars but developmental times varied between the species from 5 to 12 days under laboratory conditions. The methods used here for sample collection, species identification and aquaculture of sponge barnacle and their host described in Yu et al. 2019.

**b. Settlement recording.**

Sponge barnacle cyprids (*Membranobalanus brachialis* and *Euacasta dofleini*) were exposed to sponge pieces (approx. 15 cm^3^) and kept in water cases (13×9×4 cm) change 40 ml filtered seawater every 4 hours during observation time. The settlement process and metamorphosis were recorded by video camera (Lumix GH4) under a stereomicroscope (Leica M125 with lenses Leica 1.0X Plan Apo).

**c. Scanning Electron Microscope (SEM) preparation.**

Sponge barnacle cyprids were persevered in 30% ethanol. Pre-treatment with a series of graded ethanol dehydration (30%, 50%, 70%, 90%, 95%, each concentration for two hours). Sponge materials (included surface) are persevered in 95% ethanol after collection. Replace the preservation of cyprids and sponge materials with acetone then used critically point-dried with CO_2_ (Hitachi HCP-2). Cyprids, sponge materials and sponge spicules were sputter-coated with gold (Hitachi E-1010), and observed under SEM (FEI Quanta 200).

**d. Molecular analysis and phylogenetic reconstruction.**

The total genomic DNA was extracted from whole larvae or adult muscle tissue by a Qiagen (Chatsworth, CA) QIAquick Tissue Kit, according to the manufacturer’s instructions. DNA sequences of three mitochondrial DNA makers, 12S, 16S ribosomal DNA and cytochrome c oxidase subunit I (COI), and two nuclear markers, 18S ribosomal DNA (18S) and histone H3 (H3) were obtained to reconstruct the phylogenetic relationships. These markers have been widely used for barnacle phylogenetic analyses (Pérez-Losada et al., 2014; Chan et al., 2017; Lin e al., 2015; Tsang et al., 2014). We modified the primers and annealing temperature of the polymerase chain reaction (PCR) as described in Yu et al., 2019 and Chan et al., 2017. Direct sequencing of the purified PCR products was performed using the ABI 3730XL Genetic Analyzer with BigDye terminator cycle sequencing reagents (Applied Biosystems, Foster City, CA, USA). Sequences were assembled, edited and aligned with MUSCLE using the default parameter settings and then checked by eye in Geneious (Drummond et al., 2011).

For phylogenetic reconstruction, we collected the molecular and larval morphological data of 42 species. 18 sponge-associated barnacle species: 15 in subfamily Acastinae, 2 in subfamily Archaeobalaninae and 1 in family Pyrgomatidae; 7 coral-associated barnacle species and 13 free-living species in superorder Thoracica; 4 outgroup species: 3 in superorder Acrothracica and 1 in superorder Rhizocephala.

Bayesian inference (BI) and maximum likelihood (ML) were conducted to reconstruct the phylogenetic relationships. We used jModeltest 2.1.7 (Darriba et al., 2012) and ModelFinder (Kalyaanamoorthy et al., 2017) to determine the best-fit evolutionary model for each partition. The Akaike Information Criterion (AIC) (Akaike, 1974) is implemented. Bayesian analysis was conducted with MrBayes v.3.2.1 (Ronquist et al., 2012) with six runs carried out with four differentially heated Metropolis coupled Monte Carlo Markov Chains for 10 million generations started from a random tree. Model parameters were estimated during the analysis and chains were sampled every 1000 generations. The sampled parameter values from Bayesian MCMC were evaluated in Tracer v1.7 (Rambaut et al., 2018) and the generations before reaching a plateau were discarded as burn-in. The posterior probabilities (PP) were calculated and presented using the majority rule consensus tree. ML analyses were performed with IQ-TREE v. 1.6.8 (Nguyen et al., 2014). To assess branch support, all IQ-TREE analyses used the ultrafast bootstrap approximation (UFboot) with 1000 replicates. The parameters are using for generating BI tree and ML tree analyses are described in file [electronic supplementary material, table S1*c*,*d*].

We employed RASP 3.0 (Yu et al., 2015) for ancestral state reconstruction, the shape of attachment disc on the third segments of cypris antennules as the morphological traits for larval morphological character mapping. States of the characters were unordered and equally weighted. RASP is based on a Bayesian ancestral state reconstruction method that determines the probability of each ancestral shape-averaged over all sampled trees in the shapes of the attachment disc. We coded each taxon according to the shapes of the attachment disc. We ran 4 chains for 10 million generations in RASP, sampling every 1000th generation with a burn-in of 2.5 million generations, remove the outgroup option. The analysis was repeated for all major nodes in our molecular phylogeny tree.

**References**

Akaike H. 1974. Stochastic theory of minimal realization. *IEEE Trans*. *Autom*. Control, **19**, 667–674. (doi: 10.1109/TAC.1974.1100707)

Chan BKK, Corbari L, Rodriguez Moreno PA, Tsang LM. 2017 Molecular phylogeny of the lower acorn barnacle families (Bathylasmatidae, Chionelasmatidae, Pachylasmatidae and Waikalasmatidae) (Cirripedia: Balanomorpha) with evidence for revisions in family classification. *Zool*. *J*. *Linn*. *Soc*. **180**, 542–555. (doi:10.1093/zoolinnean/zlw005)

Darriba D, Taboada GL, Doallo R, Posada D. 2012 jModelTest 2: more models, new heuristics and parallel computing. *Nat. methods* **9**, 772. (doi:10.1038/nmeth.2109)

Drummond AJ, Ashton B, Buxton S, Cheung M, CooperA, Duran C, Field M, Heled J, Kearse M, Markowitz S. 2011 Geneious V 7.1. 4. Created by Biomatters. (https://www.geneious.com)

Kalyaanamoorthy S, Minh BQ, Wong TK, von Haeseler A, Jermiin LS. 2017 ModelFinder: fast model selection for accurate phylogenetic estimates. *Nat*. *methods* **14**, 587. (doi:10.1038/nmeth.4285)

Lin H-C, Høeg JT, Yusa Y, Chan BKK. 2015 The origins and evolution of dwarf males and habitat use in thoracican barnacles. *Mol*. *Phylogenet*. *Evol*. **91**, 1–11. (doi:10.1016/j.ympev.2015.04.026)

Nguyen L-T, Schmidt HA, von Haeseler A, Minh BQ. 2014 IQ-TREE: a fast and effective stochastic algorithm for estimating maximum-likelihood phylogenies. *Mol*. *Biol*. *Evol*. **32**, 268-274. (doi:10.1093/molbev/msu300)

Pérez-Losada M, Høeg JT, Simon-Blecher N, Achituv Y, Jones D, Crandall KA. 2014 Molecular phylogeny, systematics and morphological evolution of the acorn barnacles (Thoracica: Sessilia: Balanomorpha). *Mol. Phylogenet. Evol.* **81**, 147–158. (doi:10.1016/j.ympev.2014.09.013.)

Rambaut A, Drummond AJ, Xie D, Baele G, Suchard MA. 2018 Posterior summarisation in Bayesian phylogenetics using Tracer 1.7. Syst. Biol. syy032. (doi:10.1093/sysbio/syy032)

Ronquist F, Teslenko M, van der Mark P, Ayres DL, Darling A, Höhna S, Larget B, Liu L, Suchard MA, Huelsenbeck JP. 2012 MrBayes 3.2: efficient Bayesian phylogenetic inference and model choice across a large model space. *Syst*. *Biol*. **61**, 539–542. (doi: 10.1093/sysbio/sys029)

Tsang LM, Chu KH, Nozawa Y, Chan BKK. 2014 Morphological and host specificity evolution in coral symbiont barnacles (Balanomorpha: Pyrgomatidae) inferred from a multi-locus phylogeny. *Mol*. *Phylogenet*. *Evol*. **77**, 11–22. (doi:10.1016/j.ympev.2014.03.002)

Yu M-C, Kolbasov GA, Høeg JT, Chan BKK. 2019 Crustacean-sponge symbiosis: collecting and maintaining sponge-inhabiting barnacles (Cirripedia: Thoracica: Acastinae) for studies on host specificity and larval biology. *J*. *Crustac*. *Biol*. **39**, 522–532. (doi:10.1093/jcbiol/ruz025)

Yu Y, Harris AJ, Blair C, He X. 2015 RASP (Reconstruct Ancestral State in Phylogenies): A tool for historical biogeography. *Mol. Phylogenet. Evol*. **87**, 46–49. (doi:10.1016/j.ympev.2015.03.008)
